# The Impact of MMP-2 and Its Specific Inhibitor TIMP-2 Expression on the WHO Grade and Prognosis of Gliomas in Chinese Population: a Meta-Analysis

**DOI:** 10.1007/s12035-015-9539-x

**Published:** 2016-01-04

**Authors:** Guo-zhong Yi, Wen-yan Feng, Qiang Zhou, Ya-wei Liu, Song-tao Qi

**Affiliations:** 1 Department of Neurosurgery, Nanfang Hospital, Southern Medical University, Avenue North Road No.1838, Guangzhou, 510515 People’s Republic of China; 2 The Second College of Clinical Medicine, Southern Medical University, Avenue North Road No.1838, Guangzhou, 510515 People’s Republic of China

**Keywords:** MMP-2, TIMP-2, Gliomas, WHO grade, Prognosis, Meta-analysis

## Abstract

So far, the prognostic value of matrix metalloproteinase 2 (MMP-2) and tissue inhibitor of matrix metalloproteinase 2 (TIMP-2) expressions in patients with gliomas has been widely reported, especially in China. But, the results were inconsistent. Thus, we conducted a meta-analysis to determine the correlation of MMP-2 and TIMP-2 expressions with the prognosis of patients with gliomas. Identical search strategies were used to search relevant literature in electronic databases updated to May 1, 2015, and odds ratios (ORs) with 95 % confidence intervals (95 % CIs) were estimated. Funnel plots and Egger’s tests were conducted for the evaluation of publication bias, and heterogeneity and sensitivity were also analyzed. Finally, a total of 25 studies involving 1572 patients were included in the meta-analysis. Coincidentally, all these studies were conducted in Chinese population. It was found that MMP-2 expression was significantly associated with high-WHO grade gliomas (*n* = 24, OR = 6.54, CI = 4.98–8.60; *I* ^2^ = 0 %, *P* = 0.911) and poor overall survival (OS), while it did not correlate to age (*n* = 2, OR = 0.78, CI = 0.35–1.74; *I* ^2^ = 0 %, *P* = 0.621) and gender (*n* = 2, OR = 1.15, CI = 0.51–2.62; *I* ^2^ = 0 %, *P* = 0.995). Moreover, the results of the pooled analysis indicated that there was no association between TIMP-2 expression and the WHO grade of gliomas (*n* = 7, OR = 1.02, 95 % CI = 0.68–1.54; *I* ^2^ = 71.4 %, *P* = 0.002), but the ratio of MMP-2 and TIMP-2 (MMP-2/TIMP-2) rose with the increase of the WHO grade of gliomas. In conclusion, there was no correlation between TIMP-2 expression and the WHO grade of gliomas, while MMP-2 expression was potently associated with high-WHO grade of gliomas.

## Introduction

Gliomas are the most common primary central nervous system tumor, whose treatment mainly comprises surgical resection, postoperative radiotherapy, and chemotherapy [1, 2]. According to the World Health Organization, gliomas are divided into four clinical grades (I–IV) based on the morphology of histopathology [3]. While low-grade gliomas (I–II) are well differentiated, high-grade gliomas (III–IV) are undifferentiated and carry a worse prognosis [4, 5]. Thus, it is indispensable to identify precise biomarkers with predictive value for the grading of gliomas and identification of the survival status of patients.

Matrix metalloproteinases (MMPs) are a family of enzymes which play a direct role in the tumorigenesis process [6]. Type IV collagen is the main component of extracellular matrix (ECM) and basement membrane which form the first vital barrier in the course of tumor metastasis. Matrix metalloproteinase 2 (MMP-2), a main member of MMPs, by its ability to degrade the basement membrane type IV collagen, is thought to play a role in stromal and vascular invasion by tumor cells [7, 8]. Van Meter et al. reported that tissue inhibitors of MMPs (TIMPs) can block the action of MMPs and significantly decrease invasiveness of tumor [9]. And, TIMP-2 which is a member of TIMPs has a unique dual function that being also an inhibitor of MMPs and with a more effective action on MMP-2 [10]. The levels of both MMP-2 and TIMP-2 expressions in tumors may facilitate the initiation and progression of multiple biological behaviors required for tumor progression [10]. It has been identified that the expression of MMP-2 is associated with breast cancer [11], laryngeal cancer [12], and gastric cancer [13]. And, the amounts of TIMP-2 expression have also been found to be associated with different types of cancer [14, 15].

However, the results of studies on the association between MMP-2 or TIMP-2 expression and brain tumors were still contrary and there even existed conflicts. Some studies reported that high TIMP-2 expression was associated with high-grade gliomas [16], but some studies gave the contrary results [17], and others even showed that TIMP-2 expression was not correlated with the WHO grade of gliomas [18, 19]. Similarly, a number of studies investigated the impact of MMP-2 expression on the prognosis of patients with gliomas, but there were also no consistent results. In order to evaluate the essential roles and clinical utilities of MMP-2 and TIMP-2 in gliomas, we performed a meta-analysis.

## Materials and Methods

### Search Strategy

A literature search was carried out using PubMed, Ovid, EMBASE, Web of Science, China National Knowledge Infrastructure (CNKI), and Wanfang database updated to May 1, 2015. The search strategy used the following terms: (“metalloproteinase 2” or “MMP-2” or “type IV collagenase” or “gelatinase-A”), (“ tissue inhibitor of metalloproteinase 2” or “TIMP-2”), “gliomas [MeSH],” and “prognosis.” When the data reported in the articles were not enough for the analysis, the authors of articles were contacted. And, all references in retrieved articles were scanned to identify other potentially available studies.

### Inclusion and Exclusion Criteria

Two reviewers (GZ. Yi and WY. Feng) independently selected eligible studies. Disagreements between the two reviewers were settled by discussion with the third reviewer (Q. Zhou). The inclusion criteria were as follows: (1) studies about MMP-2 or TIMP-2 expression in patients with gliomas; (2) case control or cohort studies; (3) the main outcome of studies concentrated on WHO grade, age, gender, and overall survival; (4) MMP-2 and TIMP-2 expression which was measured by immunohistochemistry (IHC); and (5) the hazard ratio (HR) or odds ratio (OR) value which could be obtained from the article directly.

Studies were excluded based on any of the following criteria: (1) review, editorial letters, comments, or nonhuman research and (2) lacked key information for HR or OR estimation analysis. For duplicated articles, only the one with the largest sample size or the most recent study was selected.

### Data Extraction and Quality Assessment

The following information was gathered: the first author’s name, year of publication, numbers of patients, mean age, WHO grade, MMP-2 and TIMP-2 expression assay methods, cutoff value of positive expression, and follow-up duration. Relevant data were extracted by two reviewers (GZ. Yi and WY. Feng) independently, and disagreement was resolved by the third reviewer (Q. Zhou).

The quality assessment of the included studies was conducted using the Newcastle-Ottawa Scale (NOS) with some modifications [20] (Table 1), which allowed for assessment of patient population and selection, comparability, and outcome. Total NOS scores ranged from 0 to 9 with a score ≥6 indicating good quality [21].

**Table 1:** Newcastle-Ottawa quality assessment scale

Selection
1. Representativeness of the exposed cohort
(a) Truly representative of the average glioma patients in the community*
(b) Somewhat representative of the average glioma patients in the community*
(c) Selected group of users (e.g., nurses, volunteers)
(d) No description of the derivation of the cohort 2. Selection of the non exposed cohort
(a) Drawn from the same community as the exposed cohort*
(b) drawn from a different source
(c) No description of the derivation of the nonexposed cohort
3. Ascertainment of exposure (proof of gliomas and MMP-2 or TIMP-2 measurement)
(a) Secure record (e.g., surgical records)*
(b) Structured interview*
(c) Written self-report
(d) No description
4. Demonstration that outcome of interest was not present at start of study
(a) Yes*
(b) No
Comparability
1. Comparability of cohorts on the basis of the design or analysis
(a) Study controls for recurrence or metastasis*
(b) Study controls for any additional factor (age, gender, grade, etc.)*
Outcome
1. Assessment of outcome
(a) Independent blind assessment*
(b) Record linkage*
(c) Self-report
(d) No description
2. Was follow-up long enough for outcomes to occur? (death or recurrence)
(a) Yes (24 months)*
(b) No
3. Adequacy of follow-up of cohorts
(a) Complete follow-up—all subjects accounted for*
(b) Subjects lost to follow-up unlikely to introduce bias [small number lost (25 %) follow-up or description provided of those lost]*
(c) Follow-up rate (<75 %) and no description of those lost
(d) No statement

A maximum of one star (*) can be given for each numbered item within the “selection” and “outcome” categories, while a maximum of two stars (**) can be given for “comparability”

### Statistical Analysis

Quantitative meta-analysis was carried out using STATA version 12.0. Dichotomous data were presented as relative risks (RRs) or ORs, and continuous outcomes were presented as mean difference (MD), both with 95 % confidence interval (CI). Cochrane’s *Q* test and *I* ^2^ measurement were performed to assess the heterogeneity, and heterogeneity was presented as significant when *I* ^2^ ≥ 50 % or *P* ≤ 0.10. In the absence of statistical heterogeneity, a fixed-effect model was used to pool the results; otherwise, a random-effect model was used [22]. The effects of MMP-2 or TIMP-2 expression on pathological grade, age, and gender were considered as statistically significant if the corresponding 95 % CI for each pooled OR did not overlap 1, and an observed OR >1 indicated that gliomas of high-WHO grade were associated with high MMP-2 or TIMP-2 expression.

The potential risk of publication bias was examined by visual inspection of the funnel plots and was further assessed by Egger’s linear regression test, which indicated the presence of a publication bias with *P* ≤ 0.10. Sensitivity analysis was also conducted to evaluate the validity and reliability of the primary meta-analysis.

## Results

### Characteristics of Studies

**Fig. 1:**
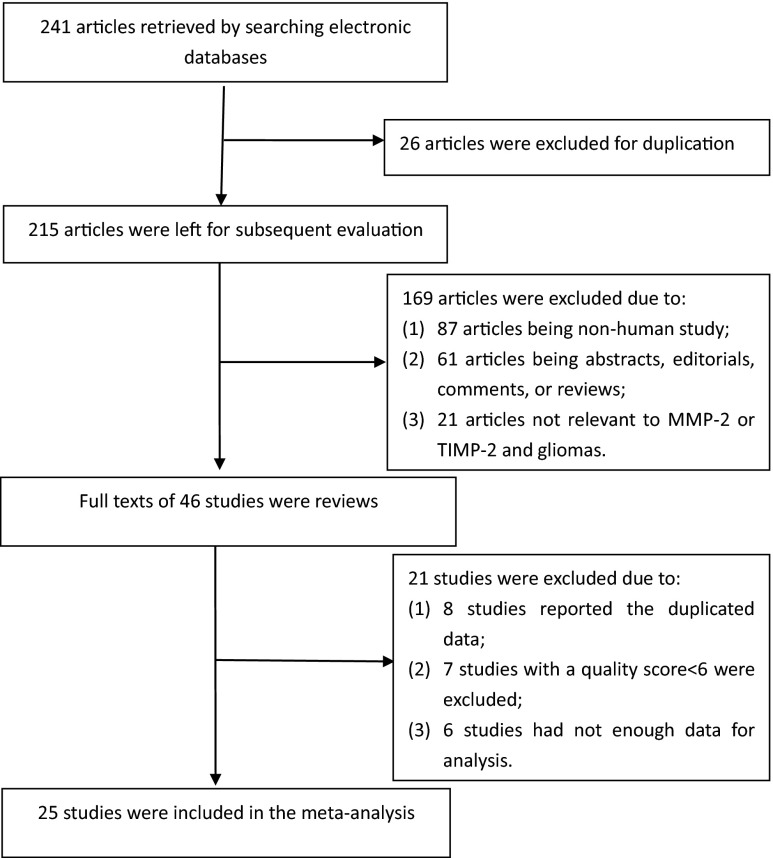
Flow chart of study selection

A total of 241 articles were identified by computer and manual search. After further reading, we excluded 216 studies according to the eligibility criteria. Finally, 25 studies were included in the meta-analysis, of which 24 studies were related to MMP-2 and gliomas and seven studies for TIMP-2 and gliomas. The flowchart of the study selection for the meta-analysis is shown in Fig. 1.

The major characteristics of all the included studies are summarized in Table 2. Interestingly, all the included studies were conducted in Chinese population, and the date of publication ranged from 2002 to 2014. Among all these studies, the correlation between the ratio of MMP-2 and TIMP-2 (MMP-2/TIMP-2) and the WHO grade of gliomas was offered in two studies [16, 39] and two studies [24, 27] provided the data of age and gender which were associated with the WHO grade of gliomas, and only one study [41] about MMP-2 expression reported the overall survival (OS) of patients. MMP-2 and TIMP-2 expressions in the tissues of gliomas were only investigated by IHC method. If the nucleus or cytoplasm was stained, the expression called local staining can be referred to as positive. For the cutoff value of positive expression, ten studies introduced the scored method according to percentage and intensity of stained cells, while the rest only used the percentage or intensity.

**Table 2:** Characteristics of included studies

Study ID	Sample size (I–II/III–IV)	Mean age	Male/female	Method	Cutoff of high expression	MMP-2 positive (I–II/III–IV)	TIMP-2 positive (I–II/III–IV)	Study quality (points)	Follow-up (months)
Sui R [23]	120 (70/50)	54.6	82/38	IHC	Score = 1	92 (46/46)	NA	7/9	NA
Zeng ZQ [24]	64 (28/36)	38.8	38/26	IHC	Score = 2	43 (15/28)	NA	6/9	NA
Guo GH [25]	40 (23/17)	45.9	26/14	IHC	5 %	24 (9/15)	NA	6/9	NA
Wu HF [26]	45 (20/25)	42.3	31/14	IHC	10 %	21 (5/16)	NA	6/9	NA
Pan LK [27]	50 (24/26)	46.3	32/18	IHC	10 %	37 (14/23)	NA	6/9	NA
Li B [18]	52 (32/20)	47.6	34/18	IHC	10 %	22 (9/13)	23 (15/8)	6/9	NA
Jv HG [16]	78 (43/35)	42.4	48/30	IHC	Score = 1	50 (21/29)	46 (21/25)	6/9	NA
Liu Q [28]	60 (27/33)	40.2	32/28	IHC	Score = 2	49 (19/30)	NA	7/9	NA
Sun SW [19]	45 (20/25)	40.2	24/21	IHC	Score = 2	36 (13/23)	24 (9/15)	6/9	NA
Zhao YF [29]	50 (17/33)	40.0	28/22	IHC	10 %	34 (8/26)	NA	6/9	NA
Song LJ [30]	100 (47/53)	42.3	58/42	IHC	Score = 1	76 (24/52)	NA	6/9	NA
Kong LF [31]	135 (64/71)	34.8	76/59	IHC	5 %	49 (11/38)	NA	6/9	NA
Lv ZH [32]	30 (12/18)	41.3	12/18	IHC	10 %	17 (3/14)	NA	6/9	NA
Zhou R [17]	67 (27/40)	43.1	40/27	IHC	10 %	41 (10/31)	41 (22/19)	6/9	NA
Li Hao [33]	50 (23/27)	48.7	28/22	IHC	Local staining	39 (13/26)	NA	6/9	NA
Li Hong [34]	119 (55/64)	42.0	66/53	IHC	Score = 1	NA	106 (45/61)	7/9	NA
Wang YT [35]	45 (20/25)	40.2	21/22	IHC	Score = 1	36 (13/23)	NA	6/9	NA
Liu ZL [36]	50 (16/34)	40.4	27/23	IHC	Score = 1	46 (13/33)	NA	6/9	NA
Tan YL [37]	68 (33/35)	38.1	37/31	IHC	Score = 1	49 (16/33)	NA	7/9	NA
Shi QH [38]	46 (21/25)	42.5	26/20	IHC	Local staining	28 (8/20)	NA	6/9	NA
Yi ZQ [39]	46 (22/24)	38.5	26/20	IHC	5 %	37 (14/23)	17 (8/9)	7/9	NA
Li J [40]	46 (30/16)	38.5	30/16	IHC	5 %	20 (7/13)	NA	6/9	NA
Xiao QH [41]	60 (20/40)	35.0	42/18	IHC	5 %	15 (1/14)	NA	8/9	>24 M
Luo GC [42] Fu XW	56 (25/31)	34.8	33/23	IHC	5 %	47 (18/29)	42 (22/20)	6/9	NA
[43]	50 (25/25)	48.5	27/23	IHC	25 %	22 (8/14)	NA	6/9	NA

Tumor grade was described on the basis of the World Health Organization (WHO) grading system of primary brain tumors and divided into two groups: low grade (I–II) and high grade (III–IV)

*IHC* immunohistochemistry, *NA* not available

### MMP-2 Expression and Glioma Grade

**Fig. 2:**
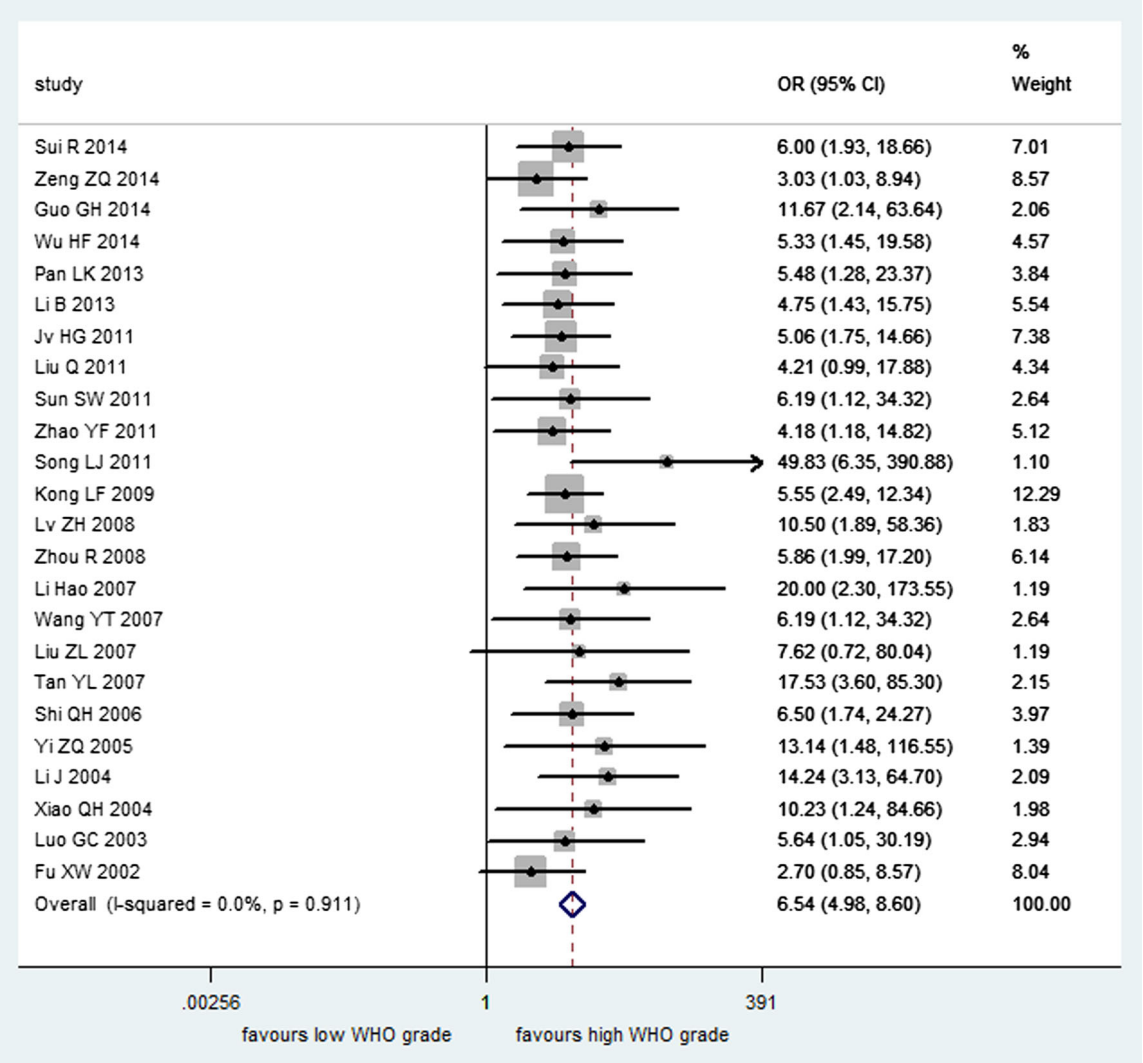
Forest plot of association between MMP-2 expression and the WHO grade of gliomas

**Fig. 3:**
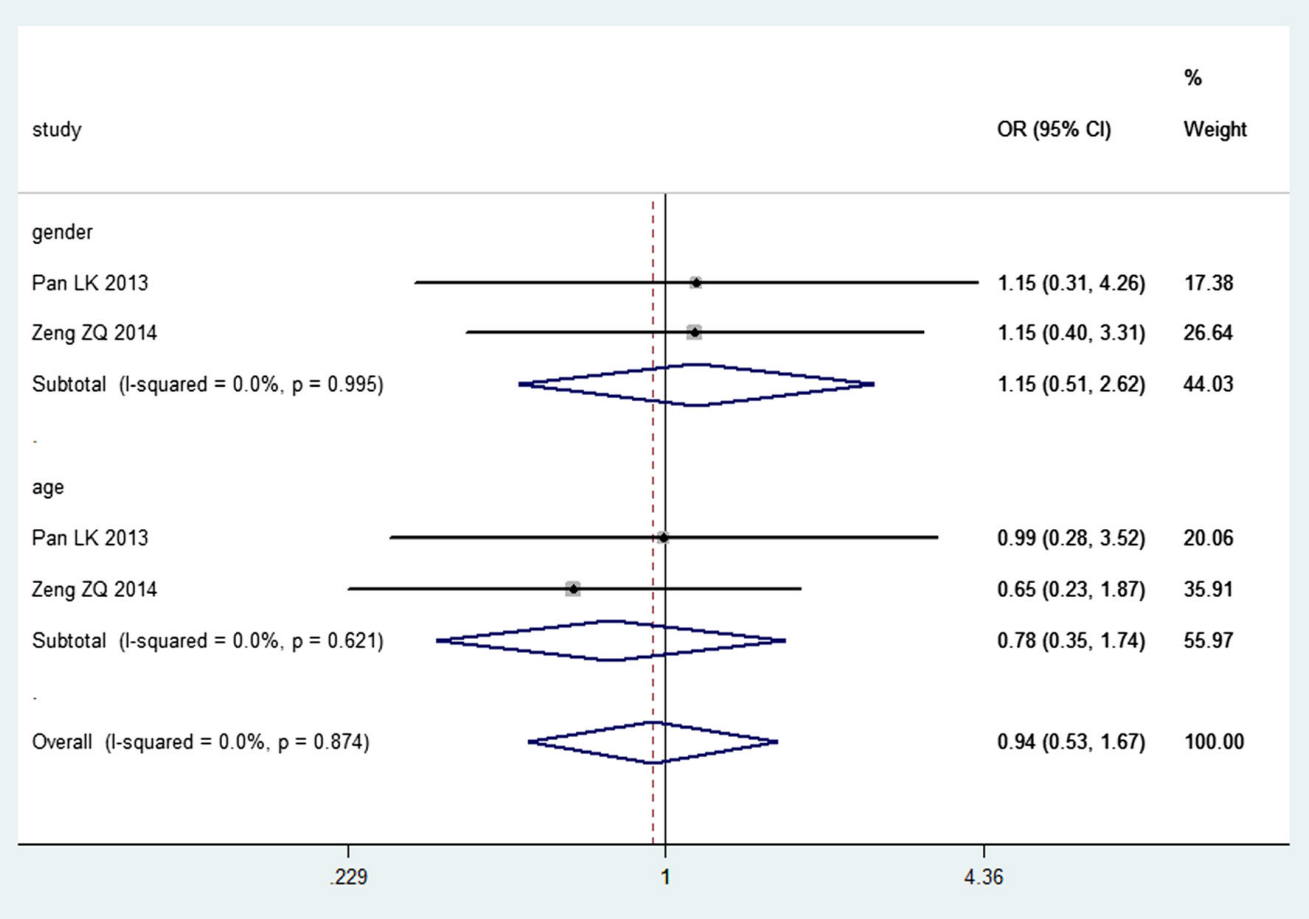
Forest plot of association between MMP-2 expression and gender, age

A total of 24 studies involving 1453 patients contained sufficient data for analyzing the association of MMP-2 expression with different WHO grades of gliomas. It was found that MMP-2 expression in patients with high-grade gliomas was significantly higher than that in patients with low-grade gliomas (*n* = 24, OR = 6.54, CI = 4.98–8.60), and there was no significant heterogeneity in the meta-analysis (*I* ^2^ = 0 %, *P* = 0.911) (Fig. 2). And, no correlation was observed between MMP-2 expression and age (*n* = 2, OR = 0.78, CI = 0.35–1.74; *I* ^2^ = 0 %, *P* = 0.621) and also between MMP-2 and gender (*n* = 2, OR = 1.15, CI = 0.51–2.62; *I* ^2^ = 0 %, *P* = 0.995) through a fixed-effect model (Fig. 3). The association of MMP-2 expression with overall survival (OS) was reported in one study [41], and the results showed that high MMP-2 expression was significantly associated with poor OS, while the exact data were not reported.

### TIMP-2 Expression and Glioma Grade

**Fig. 4:**
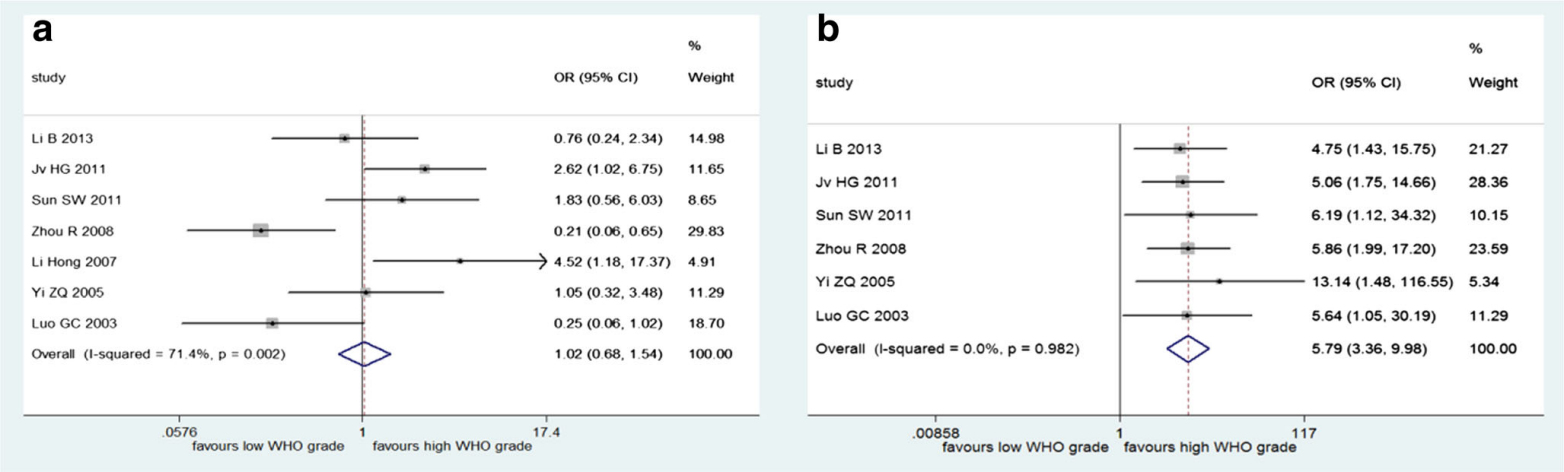
Forest plot of association between TIMP-2 expression and the WHO grade of gliomas (**a**) and association between MMP-2 expression and glioma grade in studies which have also reported TIMP-2 expression (**b**)

There were seven studies involving 463 patients on TIMP-2 expression and the WHO grade of gliomas. The results of pooled analysis demonstrated that there was no significant association between TIMP-2 expression and tumor grade (*n* = 7, OR = 1.02, 95 % CI = 0.68–1.54) (Fig. 4a), but there was significant heterogeneity (*I* ^2^ = 71.4 %, *P* = 0.002), so we performed the analysis through a random-effect model. And, among these seven studies involved, there were six studies that also reported MMP-2 expression and glioma grade, so we also conducted a meta-analysis to investigate the association between MMP-2 expression and glioma grade among these six studies. However, the overall trend was not changed (*n* = 6, OR = 5.79, 95 % CI = 3.36–9.98; *I* ^2^ = 0 %, *P* = 0.982) (Fig. 4b).

### MMP-2/TIMP-2 and Glioma Grade

Two studies reported the association between MMP-2/TIMP-2 and the WHO grade of gliomas. Of which, one study [16] used the percentage of positive cells after IHC to calculate the ratio, and the results showed that with the increase of tumor grade, MMP-2/TIMP-2 rose from 1.00 to 1.18. The other study [39] detected the gray value of microscopic field (10 × 40) and calculated the ratio, which the reported ratio is (0.83 ± 0.046) for low-grade gliomas and (0.46 ± 0.094) for high-grade tumor, while a high gray value represented the lower percentage of positive cells.

### Publication Bias and Sensitivity Analysis

**Fig. 5:**
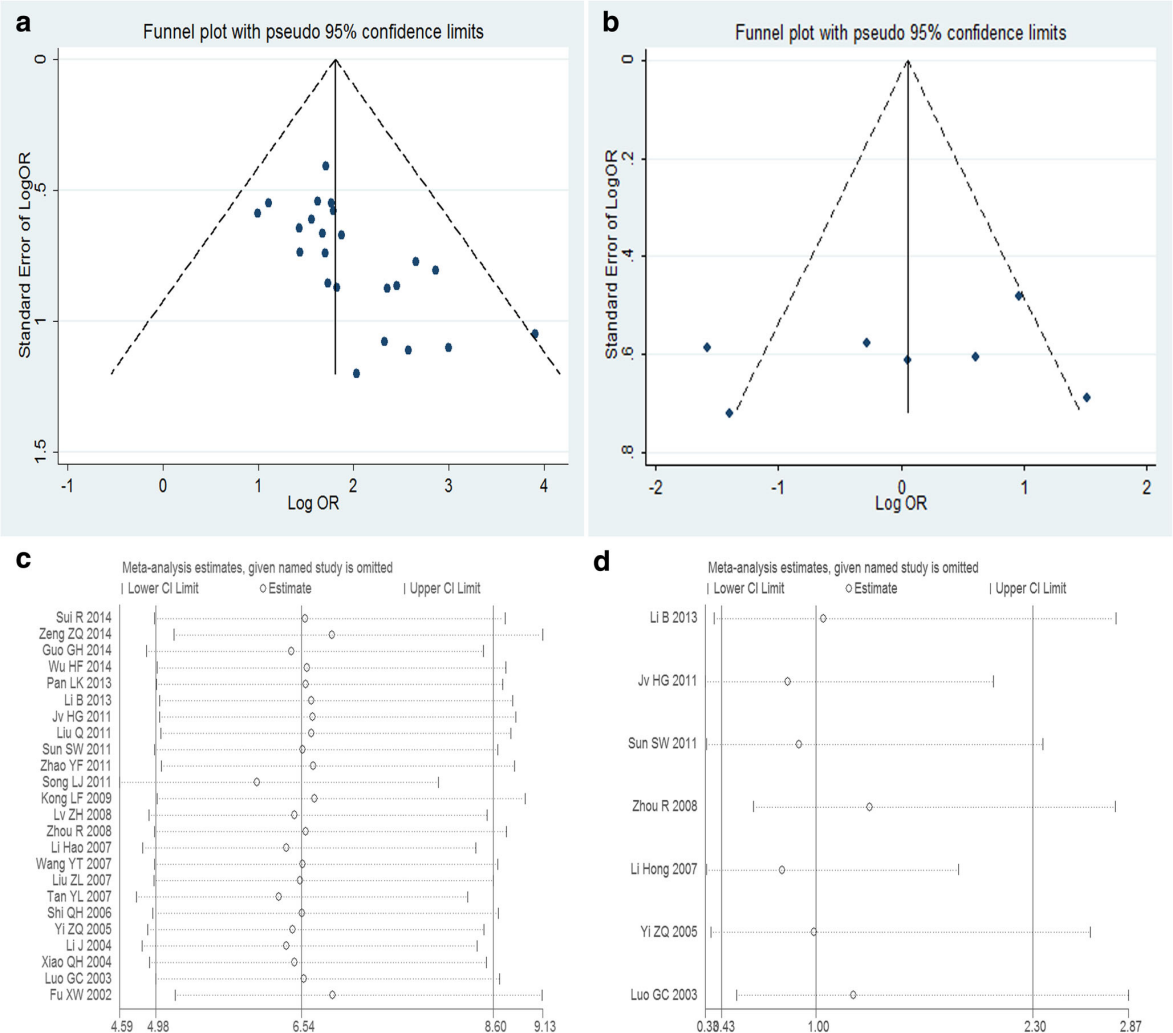
Funnel plots and sensitivity analysis of the meta-analysis. Funnel plots of the meta-analysis assessing **a** MMP-2 expression and glioma grade and **b** TIMP-2 expression and glioma grade and sensitivity analysis of the meta-analysis assessing **c** MMP-2 expression and glioma grade and **d** TIMP-2 expression and glioma grade

Visual inspection of the funnel plot revealed asymmetry in analysis of the association between MMP-2 or TIMP-2 expression and glioma grade, indicating the possibility of publication bias (Fig. 5a, b). We also estimated the publication bias through using Egger’s linear regression test. The results revealed support for significant publication bias in MMP-2 group (*t* = 3.90, *P* = 0.001) while did not reveal any publication bias among studies on TIMP-2 expression and glioma grade (*t* = −0.69, *P* = 0.521).

Sensitivity analyses were also conducted to ascertain the effects attributable to any individual study, while the results showed that no individual study could change the overall trends. It suggested that the results of the meta-analysis were stable (Fig. 5c, d).

## Discussion

Gliomas are the most common type of primary cerebral tumors. According to the WHO, gliomas can be divided into four clinical grades [3]. However, the molecular functions in glioma grade are still not wholly understood, and the prognoses of patients with gliomas remain dismal [44]. It is important for us to find the molecular markers for the progression and prognosis of gliomas, which would be of great benefit in selecting the therapeutic strategies and improving patients’ survival. Many studies have shown that MMP-2 and TIMP-2 play crucial roles in various human cancers, including gliomas. But, all the results of these studies about gliomas remained controversial, so it is necessary to perform a meta-analysis.

In our meta-analysis, the results of 24 studies involving 1453 patients demonstrated that the expression of MMP-2 in patients with high-grade gliomas increased significantly as compared to patients with low-grade gliomas, while no correlation was observed between MMP-2 and age, and also no association was found between MMP-2 and gender. There were also evidences that high MMP-2 expression was associated with poor OS in patients with gliomas. Moreover, our meta-analysis determined that there was no association between TIMP-2 expression and the WHO grade of gliomas, and the overall trend of MMP-2 expression and glioma grade was not changed in studies which have reported both MMP-2 and TIMP-2 expressions. And, MMP-2/TIMP-2 rose with the increase of glioma grade, which may be used as criteria of WHO grade in gliomas.

Both funnel plots and Egger’s tests indicated that publication biases were present in the analysis of association of MMP-2 expressions with glioma grade, though we performed a quality assessment of studies to avoid some selection biases and attempted to minimize publication bias by performing the literature search as complete as possible. However, our search was restricted to studies published in English or Chinese, and the studies with negative results were often rejected [45]. So, publication bias may be a limitation in our meta-analysis.

Despite our efforts to conduct a comprehensive analysis, there were also some other limitations which should be discussed. Firstly, the heterogeneity among studies about TIMP-2 expression and glioma grade may affect the results of the present meta-analysis. Secondly, all the MMP-2 and TIMP-2 expressions were measured by IHC and all the studies were conducted in Chinese population. IHC was the most frequently applied method, but methodological differences may contribute to the heterogeneity. And, subgroup analysis could not be conducted to address these technical problems because few studies offered the concrete data. Thirdly, there was no consistent threshold value to define positive expression in assessment of MMP-2 and TIMP-2 expressions, which can be the cause of potential bias. Lastly, there was only one study that reported the data about MMP-2 expression and the OS and only two studies analyzed the association between MMP-2/TIMP-2 and the grade of gliomas. Due to the limited number of studies included, the findings about the impact of MMP-2 expression or MMP-2/TIMP-2 on the prognosis of gliomas should be interpreted with caution.

Considering all these limitations existing in this meta-analysis, the conclusions of this meta-analysis should be drawn carefully. In summary, our meta-analysis concluded that high MMP-2 expression was associated with high-grade gliomas, and there was no correlation between TIMP-2 expression and the WHO grade of gliomas. MMP-2/TIMP-2 testing may predict the WHO grade of gliomas, and MMP-2 expression may serve as a biomarker for the prognosis of patients with gliomas, which required to be further certified by future studies.
